# Morphometric analysis of patella and patellar ligament: a cadaveric study to aid patellar tendon grafts

**DOI:** 10.1007/s00276-021-02837-z

**Published:** 2021-09-27

**Authors:** Ashwini Aithal Padur, Naveen Kumar, Melissa Glenda Lewis, Varalakshmi Chandra Sekaran

**Affiliations:** 1 Department of Anatomy, Melaka Manipal Medical College (Manipal Campus), Manipal Academy of Higher Education (MAHE), Manipal, Karnataka 576104 India; 2 Indian Institute of Public Health, Shillong, Meghalaya India; 3 Department of Community Medicine, Melaka Manipal Medical College (Manipal Campus), Manipal Academy of Higher Education (MAHE), Manipal, Karnataka India

**Keywords:** Patella, Patellar tendon, Dimensions, Morphometric, Tendon graft

## Abstract

**Purpose:**

Morphometric analysis of the patella and the patellar ligament is crucial in diagnosing and surgical corrections of knee injuries and patellofemoral joint disorders. Dimensions of the patella and the patellar ligament are frequently used in implant design and ACL reconstruction. This study aims to obtain detailed morphometric data on the patellar ligament and its localization based on gross anatomical dissections in the adult cadavers.

**Methods:**

The present study consisted of 50 lower limbs from formalin-fixed male adult cadavers aged about 70 years (45–85) belonging to the South Indian population. Total length of the quadriceps tendon, patellar height, patellar ligament height, proximal width, distal width and thickness of the patellar ligament were measured meticulously. Mean, standard deviation, median scores of each parameter were computed for groups using SPSS 16.0. Level of significance was considered as *p* < 0.05. Wilcoxon signed-rank test was used to compare the various parameters on the right and left limbs. The relationships between all parameters were analyzed using Spearman’s rank correlation test.

**Results:**

There was no statistically significant difference in the various measurements of the patella and patellar ligament between the right and left lower limbs. Patellar ligament length showed positive correlation with ligament thickness (*r* = 0.36; *p* = 0.078 for right limb and *r* = 0.33; *p* = 0.104 for left limb). Proximal width of ligament showed significant positive correlation with distal width (*r* = 0.41; *p* = 0.041 for right limb and *r* = 0.54; *p* = 0.006 for left limb).

**Conclusion:**

This morphometric data and analysis might be fundamental in understanding various knee conditions in situ and necessary to orthopedic surgeons for successful planning and execution for ACL reconstruction using patellar ligament graft and other patellofemoral joint disorders.

**Level of evidence:**

I

## Introduction

The most common injury at the knee joint, especially in sportspeople, is a tear of the anterior cruciate ligament (ACL). The patellar ligament and quadriceps tendon are commonly used as grafts for ACL reconstruction, surgical revision, and multi-ligament surgery to restore functional stability. The patella is the largest sesamoid bone in the body that develops within the tendon of the quadriceps femoris muscle and is found on the anterior surface of the femoral condyles. It has two surfaces (anterior and posterior), three borders (superior, medial, and lateral), and an apex pointing inferiorly [31]. The patella, also called a knee cap, does not have morphological sex determination; nevertheless, it is one of the few bones in the human body that is resistant to post-mortem changes [25]. The extensor mechanism of the knee consists of the quadriceps muscle group and tendon, the patella, the patellar ligament, the tibial condyles, and the patellar retinaculum [11]. The patellar tendon (patellar ligament) is the central band of the tendon of the quadriceps femoris, which is continued distally from the patella to the tibial tuberosity. It is strong, flat, about 6 cm in length, and is attached proximally to the patellar apex and adjoining margins, while distally, it is attached to the tibial tuberosity [31]. This insertion is oblique and directed laterally. In the procedure of tibial osteotomy, the tibia is cut transversely just above the patellar tendon insertion. Failure to appreciate the obliquity of the tibial attachment of the tendon may lead to an unintended division of the tendon during this procedure, leading to unfortunate consequences [31].

Morphometric data from the studies on the patella and patellar ligament are crucial in the diagnosis and surgical corrections of knee-related injuries [21, 33] and patellofemoral joint disorders [2, 28]. Dimensions of the patella and patellar ligament are frequently utilized in implant design and specific surgical procedures such as patella resurfacing for total knee arthroplasty and the harvesting technique of patellar ligament grafts during the reconstruction of the anterior cruciate ligament [24, 26]. Determining the relationship between the patella and patellar ligament in different population groups is essential anthropologically and clinically. Understanding the pathogenesis of disorders involving the knee calls for detailed knowledge of the normal anatomy and biomechanics of the patella and the patellar ligament [9]. Previous studies have shown significant evidence-based racial differences in the morphometry of the knee joint, patellar ligament amongst the studied populations [8]. There are not any detailed studies dealing with the patella and patellar ligament morphometric measurements using cadavers belonging to the Indian population. The advantage of the measurements performed in a cadaveric approach is that they do not involve approximation and assumptions. Thus, in this study, we aimed to obtain detailed morphometric data on the patellar ligament and its localization based on gross anatomical dissections in the adult cadavers.

## Materials and methods

**Fig. 1:**
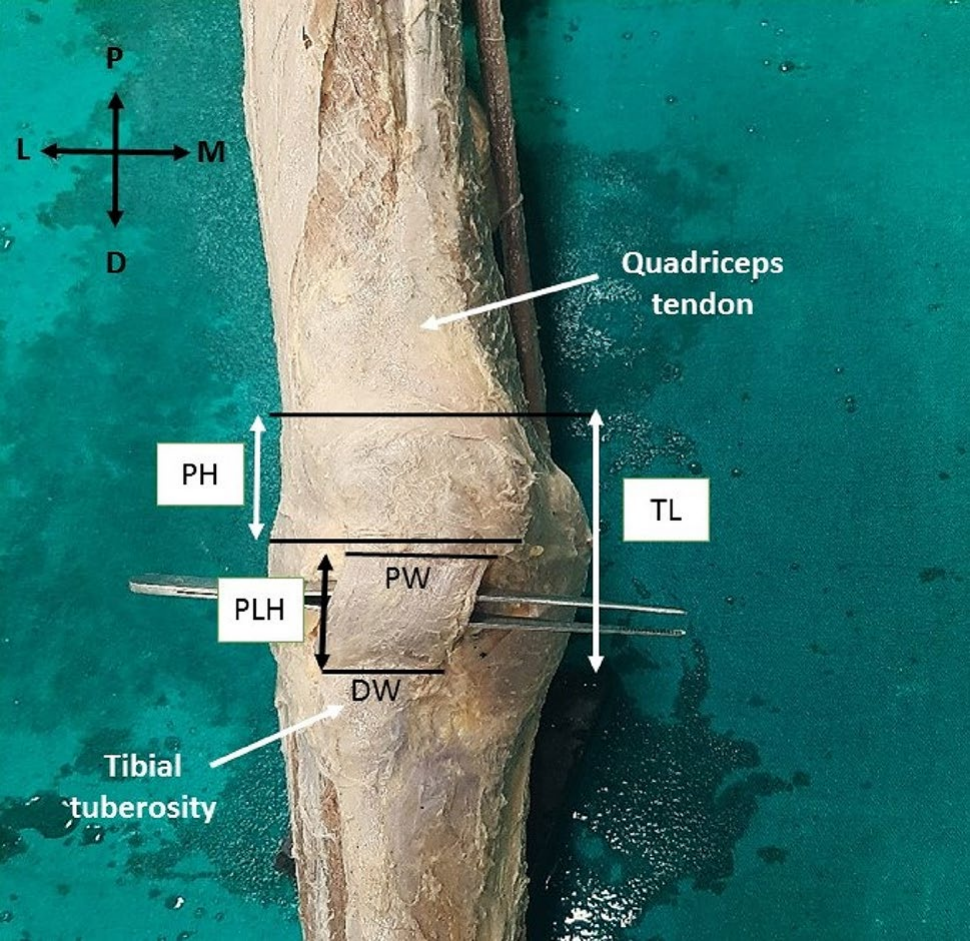
Figure showing the various measurements of the patella and patellar ligament. *TL* total length, *PH* patella height, *PLH* patellar ligament height, *PW* patellar ligament width (proximal end), *DW* patellar ligament width (distal end). Axis: *P* proximal, *D* distal, *M* medial, *L* lateral

The study sample consisted of 50 lower limbs (25 right lower limbs and 25 left lower limbs) from formalin-fixed (10% formalin) male cadavers belonging to the South Indian population, procured from the Anatomy department of our college. The mean age of the cadavers was 75 years (45–85). Cadavers with osteoarthritic changes to the knee, visible surgical scars in the knee region, physical signs of deformity of the patella, or patellofemoral disease were excluded from the study. The incision was made on the medial sides of both knees of the cadaver, skin and facia lata were carefully removed to expose the quadriceps tendon, the patella, and the patellar ligament. The tendon of the quadriceps femoris and the patellar ligament was carefully freed from the underlying structures without causing any alteration to the desired structures. All measurements were performed while the patella and patellar ligament were in situ. The various morphometric measurements were measured using a calibrated rigid ruler (accuracy, 0.5 mm) and silk suture thread. All measurements were taken twice, in the same manner by the same investigator for all the limbs to avoid interobserver variability. Since there was no differences in the measurements, intra-observer reliability test was not performed. Total length of the quadriceps tendon, patellar height, patellar ligament height (patellar tendon length), proximal width, distal width and thickness of the patellar ligament were measured meticulously. The description of the measurements is shown in Table 1 and Fig. 1.

**Table 1:** Description of measurements taken of the patella and patellar ligament

Measurement	Abbreviation	Description
Total length	TL	The linear distance between the base of the patella and the attachment of the patellar ligament to the tibial tuberosity
Patella height	PH	The linear distance between the superior border and the apex of the patella
Patellar ligament height/patellar tendon length	PLH	The linear distance between the apex of the patella and the tibial tuberosity
Patellar ligament width (proximal end)	PW	Maximum width of the ligament just below the apex of the patella
Patellar ligament width (distal end)	DW	Maximum width of the ligament just above tibial tuberosity
Patellar ligament thickness	PT	The thickness of the ligament above its distal attachment

The mean, standard deviation, median scores of each parameter were computed concerning groups using SPSS 16.0 statistical package (SPSS Inc, Chicago, IL). Level of significance (*p*-value) was set at 0.05. Wilcoxon signed-rank test was used to compare the various parameters on right and left limbs. The relationships between all parameters were analyzed using Spearman’s rank correlation test. To determine the statistical power, we performed power analysis for correlation in R version 3.6.3. The correlation power analysis estimated a power of 82.2% to determine the statistical power between the proximal width vs distal width in the left lower limb with a sample size of 25, level of significance of 5% and correlation coefficient of 0.54. Results were represented in relevant tables and graphs (Tables 2, 3, 4 and 5).

**Table 2:** Table showing the various morphometric measurements of the patella and patellar ligament

Variables	Right lower limb		Left lower limb	
	Median	Mean ± SD (cm)	Median (range)	Mean ± SD (cm)
Total length (TL)	10	9.96 ± 0.84	10	9.88 ± 0.88
Patellar height (PH)	5	5.08 ± 0.64	5	5.2 ± 0.78
Patellar ligament height (PLH)	5	4.88 ± 0.65	5	4.68 ± 0.91
Proximal width (PW)	3.5	3.56 ± 0.43	4	3.50 ± 0.44
Distal width (DW)	2.5	2.62 ± 0.49	2.5	2.48 ± 0.45
Patellar ligament thickness (PT)	6	5.92 ± 0.62	6	5.80 ± 0.74

## Results

### Side differences

**Fig. 2:**
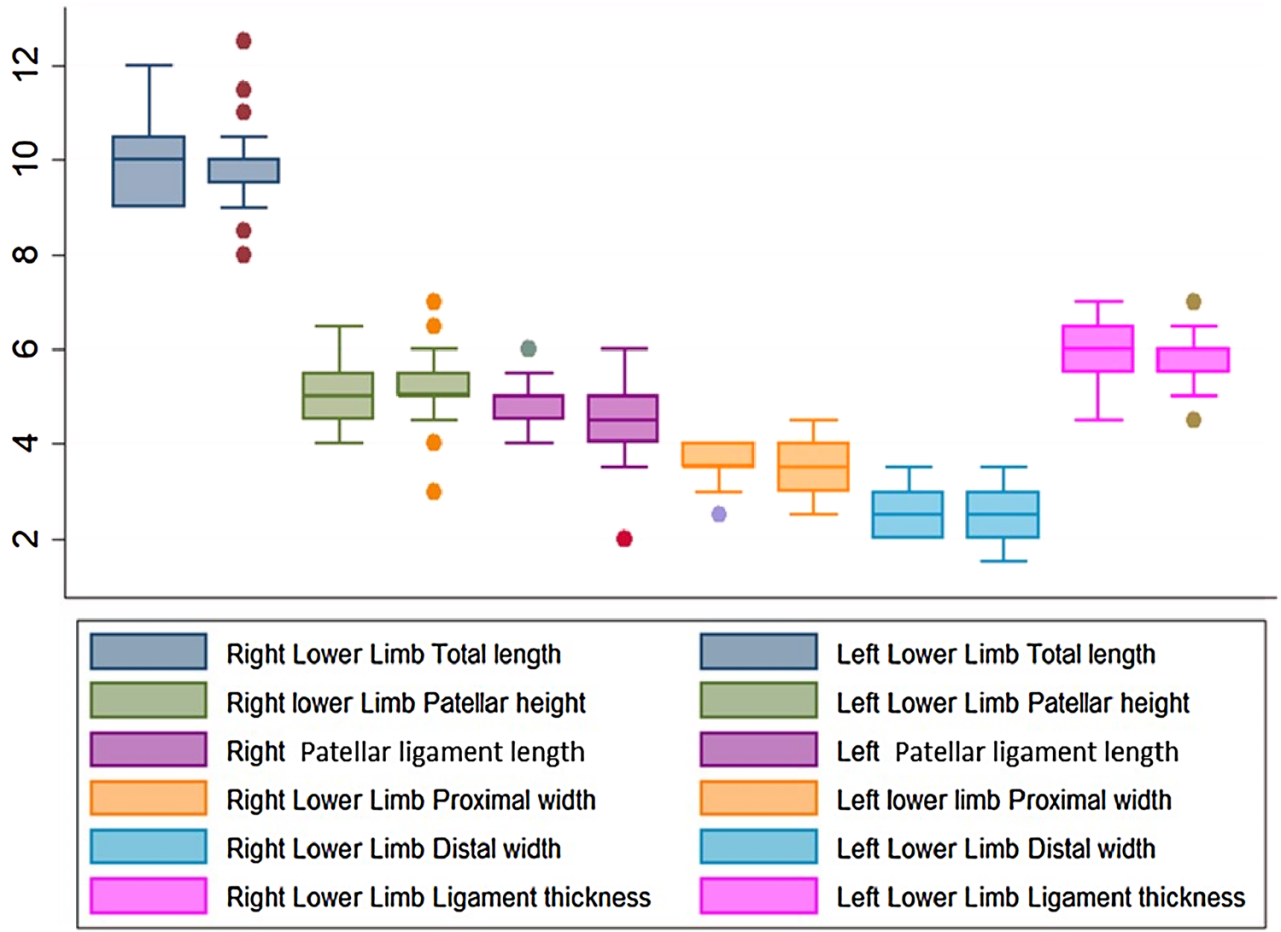
Comparison of morphometric measurements between right and left lower limbs

A Wilcoxon signed-rank test showed that there was no statistically significant difference in the various measurements of the patella and patellar ligament between the right and left lower limbs (*p* > 0.05) (Fig. 2, Table 3). The average length of the patellar ligament was 4.8 cm on the right limb and 4.6 cm on the left limb. The shortest ligament was about 2 cm (range 3–6 cm) was observed in the left limb with no obvious pathology. Patellar height was almost the same in both limbs (5.03 cm) (Table 3).

**Table 3:** Table showing the result for Wilcoxon signed-rank test to compare the level of significance between the studied variables in the right and left limbs

Wilcoxon Signed rank test	
Variables	*p*-value (between right and left limbs)
Total length (TL)	0.664
Patellar height (PH)	0.322
Patellar ligament height (PLH)	0.368
Proximal width (PW)	0.551
Distal width (DW)	0.3137
Patellar ligament thickness (PT)	0.4437

### Descriptive analyses

The mean, standard deviation, and median scores for the measurements of the patella and the patellar ligament are presented in Table 2.

### Analyses of correlation

Spearman’s correlation analysis was performed.

**Fig. 3:**
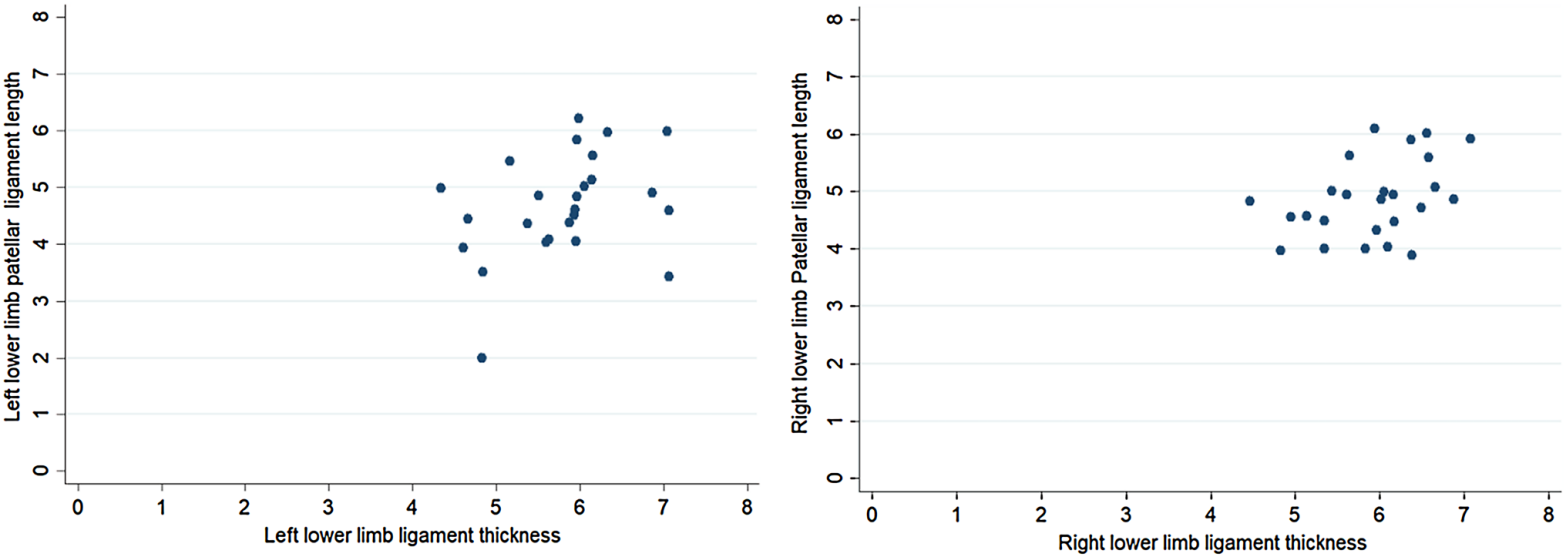
Correlation between lengths of patellar ligament vs. ligament thickness in right and left lower limbs

**Fig. 4:**
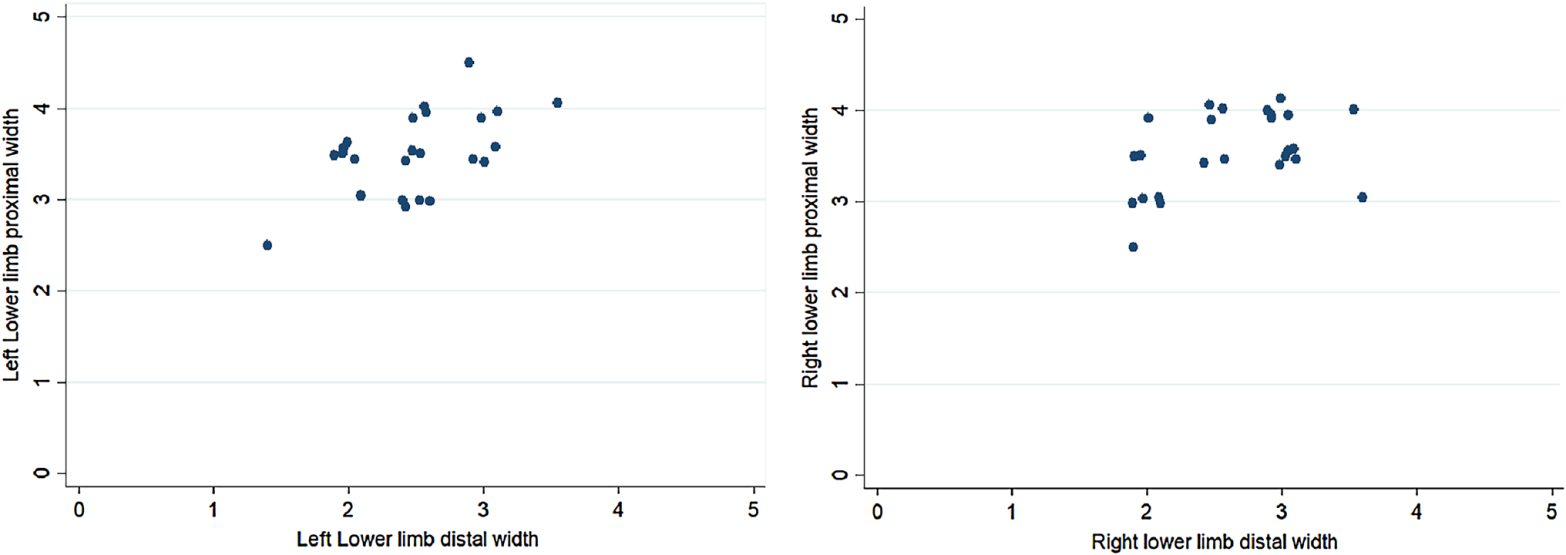
Correlation between Proximal width vs. distal width of the patellar ligament between the right and left lower limbs

**Fig. 5:**
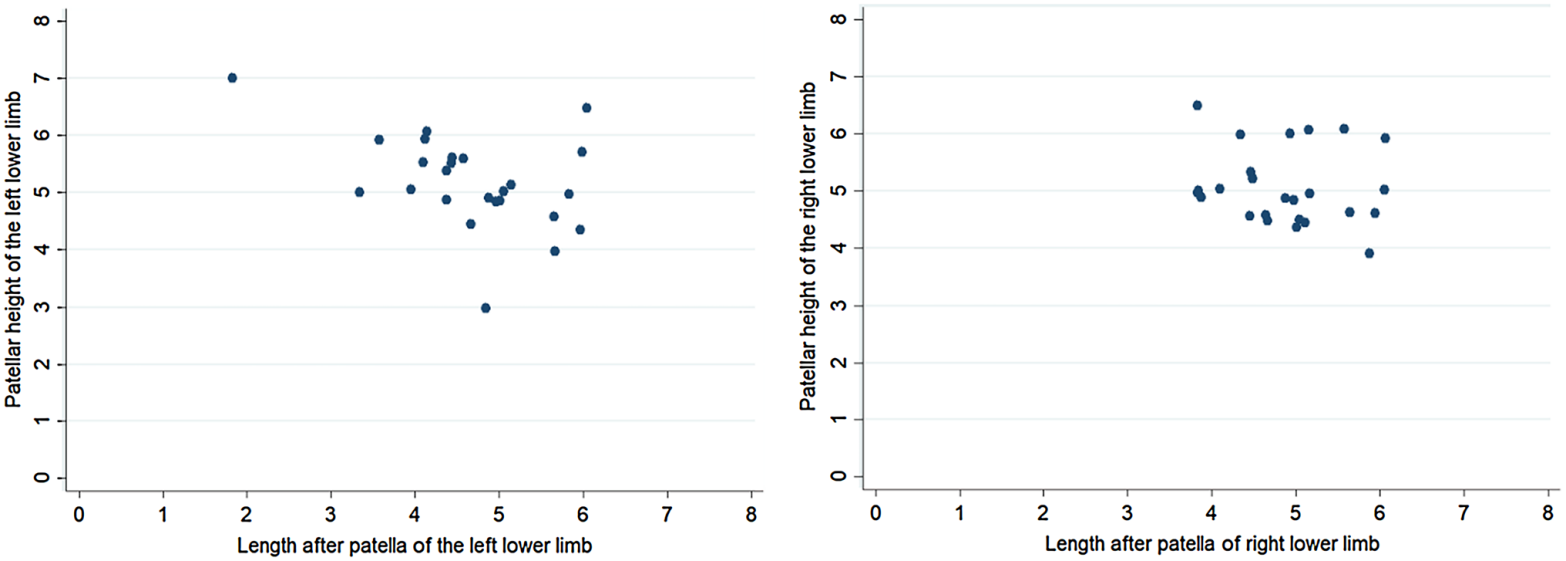
Correlation between patellar height vs. length after patella (patellar ligament height) of the right and left lower limbs

Patellar ligament length showed low correlation with ligament thickness (*r* = 0.36; *p* = 0.078 for right limb and *r* = 0.33; *p* = 0.104 for left limb) (Table 4, Fig. 3). Proximal width of ligament showed significant positive correlation with distal width (*r* = 0.41; *p* = 0.041 for right limb and *r* = 0.54; *p* = 0.006 for left limb) (Table 5, Fig. 4). There was a negative (*r* = − 0.18) statistically non-significant (*p* value = 0.38) relationship between patella height and patellar tendon length (PLH) in the right lower limb. However, in the left lower limb, there was a negative (*r* = − 0.47) statistically significant moderate (*p* value = 0.02) relationship between patella height and patellar tendon length (PLH) in the right lower limb (Fig. 5). This indicates that, as the patellar height increases, the patellar tendon length decreases.

**Table 4:** Table showing the correlation between the length of the patellar ligament and ligament thickness in the right and left lower limbs

Length of patellar ligament vs ligament thickness	Spearman’s rank correlation coefficients	*p*-value
Right lower limb	0.36	0.078
Left lower limb	0.33	0.104

**Table 5:** Table showing the correlation between proximal and distal width of the patellar ligament in the right and left lower limbs

Proximal width vs distal width of the patellar ligament	Spearman’s rank correlation coefficients	*p*-value
Right lower limb	0.41	0.041*
Left lower limb	0.54	0.006*

*At 5% level of significance

## Discussion

A relevant anatomical knowledge regarding musculoskeletal structure with its associated relationships has always been essential to orthopedic surgeons. Although many studies focus on the geometry of patella and patellar tendon, these studies are mainly radiological, intraoperative measurements, and biomechanical studies. There are very few cadaveric studies dealing with the actual in situ measurements of the patella and patellar ligament to the authors' knowledge. Thus, this study was aimed to provide the whole integrated morphometry of the patella and patellar ligament using anatomical dissection. Although the anatomical methods have the disadvantage of limited clinical material for substantial clinical research, the data obtained from these studies are beneficial for adapting to modern surgical procedures.

Morphometric measurements of the patella are important anthropologically as they can be used in forensic science for sex determination with the highest accuracy rate. In the present study mean height of the patella was approx. 5 cm on both limbs. This finding is similar to the study of Schlenzka et al., wherein the mean patellar height was 5.4 cm in 50 fresh cadaveric patellae studied by them [28]. In previous studies, the mean patellar height was found to be 44.6 mm which was MRI study [33], 39.9 mm in a study on CT scans from 40 Chinese volunteers [30], 42.96 mm in a study using dry patella [4], 38.07 mm in a study on dry patella of Indian population [20], and 43.7 mm in cadaveric patellae of European ancestry [23]. Although the reasons for variations in patellar height cannot be validated, the differences may be due to the difference in methods of measuring, age, sexes, study group, ethnicity, and stature [23]. It is known that morphometric variations are often observed in measurements even within the same population group and that the variation could be more striking across different ethnic groups.

Dimensions and classification of patellae are important for anthropologists and surgeons for the determination of the size of a patellar implant [18]. A disproportional implant of the patellofemoral joint would result in ineffective lever support, limitation of motion, excessive wear, and patella instability with associated knee pain [14].

The development of the patellar ligament in the fetal period is important as the pathologic conditions of the patellar ligament in adulthood may be associated with fetal development [5, 19]. Patellar ligament plays a vital role in the extensor mechanism of knees, and patellar tendinitis, partial or complete ligament tears, can result in loss of ligament function. It is opined that complete patellar ligament tears are primarily seen in athletes, volleyball, and basketball players [10]. Ligament tear will result in restricted activity levels, thereby decreasing quality of life [16]. Hence knowledge regarding the exact anatomy of the patellar ligament is important to understand its functional mechanism and aid in its surgical reconstruction. Another common ligament injury seen nowadays is the tear of the anterior cruciate ligament (ACL). ACL is an intracapsular ligament of the knee joint. The area of origin and insertion of the ACL is reported to average 113 and 136 mm^2^, respectively. The cross-sectional area at midsubstance varies between 36 and 44 mm^2^, while the length of the anterior and posterior aspect of the ligament is reported to vary between 22 and 41 mm [7, 12]. With varying techniques employed by surgeons, the source of the graft tissue to replace the damaged ACL is of critical importance to ensure the best outcome. The most commonly used grafts for ACL reconstruction are those of the patellar ligament and the hamstrings (semitendinosus or gracilis), to a lesser extent, the fascia lata and quadriceps tendon [27]. Although there are numerous options available, there is no agreement in the literature on the most suitable choice for the graft source [1, 17, 22]. Many factors need to be considered in selecting a graft material like, it should be easily accessible, should rapidly ligamentize once implanted, and be comparable to the strength of ACL [13]. Therefore, the patellar ligament is the most favored choice for graft as it has greater tensile strength, it may be vascular, and can have some bony part with it providing better chances for successful graft [3]. Both the quadriceps tendons and patellar ligaments have been tested for strength and load-bearing capacity and are comparable in this regard [15]. The patellar ligament is stronger and more rigid than the quadriceps tendon [29]. Patellar tendon bone grafts should be used for young patients and high demand athletes who prefer early return to high-level activities, while hamstring tendons are advantageous when a large skin incision or anterior knee pain should be avoided [6]. The length of the patellar tendon is a major concern in ACL reconstruction surgery with a patellar tendon graft. The bone peg harvested from the inferior pole of the patella is usually placed at the femoral tunnel, and that from tibial tuberosity at the tibial tunnel, and the ligament substance between the bong pegs acts as the reconstructed ACL [33].

We found in our study that the length of the patellar ligament on the right limb was slightly more when compared to the left limb, and a slight positive correlation was observed between the ligament length and ligament thickness in both the limbs (*r* = 0.36 for right limb and *r* = 0.33 for left limb). Even though we could not give exact justification for these observations, we assume that factors such as predominant use of one limb, posture, physique, etc., might produce better muscle tone on one side, thereby directly affecting the length and thickness of the patella and patellar ligament. A positive correlation was also observed between the proximal and distal width of the patellar ligament in both limbs (*r* = 0.41 for right limb and *r* = 0.54 for left limb). The patellar ligament was wider proximally than distally, which was following a previous study [32]. The authors opine that this difference is because the tendon fascicles tend to converge toward the midline before their attachment to the tibia. Although the measurements performed in a cadaveric approach is advantageous as they do not involve approximation and assumptions, the measurements obtained from images are more accurate and repeatable. A single technique is unlikely to be optimal in all circumstances. Therefore, we suggest that morphometric measurements from the cadaveric method and radiological methods could be integrated to provide optimum accuracy and desired reproducibility in clinical settings.

Limitations of the present study include the use of embalmed cadavers; the gender-wise difference could not be calculated due to the non-availability of female cadavers and the absence of data such as height or BMI of the cadavers. Although we found a correlation between patellar ligament length and thickness, we could not find detailed studies in the literature to correlate our findings and assumptions. Another limitation of the study was that, as two or more observers could not do the morphometric measurements, the inter-observer error of measurement could not be performed. Therefore the accuracy of measurements could not be reinforced.

## Conclusion

The present cadaveric study is beneficial for local anthropological records and provides an essential reference guide for designing patellar prosthetic implants for the South Indian population. This morphometric data might be fundamental in understanding various knee conditions in situ and necessary to orthopedic surgeons for successful planning and execution for ACL reconstruction using patellar ligament graft and other patellofemoral joint disorders.

## Data Availability

Data from human cadavers were used for the study.
